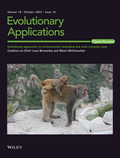# Cover Image

**DOI:** 10.1111/eva.13411

**Published:** 2023-11-21

## Abstract

Caption: An infant of Macaca mulatta vestita is groveling on adult‐female monkey's back in Jiacha Gorge on the Yarlung Zangbo River, Tibet, China.

Credit: Dayong Li.